# Influence of plant size on female-biased sex allocation in a single-flowered, nectarless herb

**DOI:** 10.1093/aobpla/plv139

**Published:** 2015-11-24

**Authors:** Ying-Ze Xiong, Meng Xie, Shuang-Quan Huang

**Affiliations:** 1 Institute of Evolution and Ecology, School of Life Sciences, Central China Normal University, Wuhan 430079, China; 2 Department of Cultural Heritage Conservation, Hubei Provincial Museum, Wuhan 430077, China

**Keywords:** Animal-pollinated, Berberidaceae, biomass, gamete production, *Podophyllum hexandrum*, single-flowered, size-dependent sex allocation

## Abstract

Relative allocation to female and male function in hermaphroditic species often departs from strict equisexuality. Increased femaleness with plant size in animal-pollinated species has been proposed in theory and demonstrated in empirical studies. However, such size-dependent sex allocation (SDS) has not been observed in some insect-pollinated species, throwing doubt on the generalization of SDS, that large plants have decelerated male function investment. Himalayan mayapple *Podophyllum hexandrum* (Berberidaceae) produces a single terminal flower and no nectar, providing a simple system for studying SDS without the confounding effects of flower number and nectar production. To investigate the SDS in *P. hexandrum*, plant size, biomass of floral organs (stamens, pistils and petals) and gamete production (pollen and ovule number) were measured in four populations in Yunnan Province, northwest China. Isometric allocation to female and male function with plant size was found in two populations, but the prediction of SDS was supported in the other two populations. Using pollen and ovule production as the allocation currency, allocation to female and male function was isometric in all studied populations. Resources allocated to attractive (petals) and sexual (pistils and stamens) structures did not show a significantly disproportionate increase with plant size in three of the four studied populations. The general pattern of isometric allocation to female and male function and to attractive and sexual structures could be attributed to the species being capable of automatic self-pollination, related to low pollen loss, minor deleterious effect of selfing and low importance of attractive structures. However, in further studies, careful consideration should be given to the different currencies used to estimate sex allocation.

## Abstract (precis)

Patterns different from size-dependent sex allocation (SDS) may provide insights into how plants modify their sex allocation in various habitats. Although an increase in female-biased sex allocation with plant size has been observed in numerous animal-pollinated species, exceptions do exist. In single-flowered, nectarless Himalayan mayapple, allocation to female and male function was proportionately increased with plant size in two of the four studied populations. The isometric allocation was evident when pollen and ovule production were measured as the allocation currency, with no biased allocation to floral attractive structures, perhaps as the species is capable of automatic self-pollination.

## Introduction

The theory of sex allocation proposes that co-sexual plants should allocate equal resources to female and male function if the fitness returns of relative investment into these two functions are equal (Charnov 1982; de Jong and Klinkhamer 2005). However, fitness returns of female and male components are often differentially affected by changes in resource status, so that organisms are expected to modify their sex allocation based on the variation in plant size (Charnov 1982; Lloyd and Bawa 1984). One may expect that a large plant would be able to allocate more available resources to produce seeds, resulting in increasing femaleness with plant size. Numerous theoretical and empirical studies suggest that relative allocation to female function increases with plant size (see Lloyd and Bawa 1984; Bickel and Freeman 1993; Klinkhamer *et al*. 1997). Such a pattern of size-dependent sex allocation (SDS) has mostly been found in animal-pollinated species (Bickel and Freeman 1993; Klinkhamer *et al*. 1997), while some wind-pollinated species showed an opposite pattern—maleness increasing with size (Bickel and Freeman 1993).

Since female function involves more resource cost to produce seeds, female-biased sex allocation with plant size should be more common under resource limitation condition (sex-differential resource costs, Lloyd and Bawa 1984; Bickel and Freeman 1993; Day and Aarssen 1997). For example, female-biased allocation increased with plant size at a shaded site while male-biased allocation increased with size at an open site in *Tussilago farfara* (Asteraceae) (Torices and Méndez 2011). In a monocarpic perennial herb *Cardiocrinum cordatum* (Liliaceae), male allocation (pollen production) decreased with plant size in a population under a closed canopy but not in the population under a sparse canopy (Cao and Kudo 2008). Thus, resource condition could play an important role in affecting relative allocation to female and male function.

Practical problems in direct measurement of the fitness returns of relative investment into male and female functions make it difficult to predict which gender will be emphasized. Different currencies have been used to estimate resource allocation to flowers, such as biomass, flower (gamete) number and nutrient (nitrogen and phosphorus) content, but may yield different allocation patterns (Méndez and Traveset 2003; Torices and Méndez 2011). It has been suggested that sexual allocation should be assessed in several currencies to the greatest degree possible (Goldman and Willson 1986; Charlesworth and Morgan 1991).

Himalayan mayapple *Podophyllum hexandrum* (Berberidaceae) is a suitable system on which to test SDS. This spring flowering herb usually produces a single terminal flower before leaf emergence, so that potential effects of plant size acting through leaves are excluded. In this single-flowered species, it is only the size of floral organs that is modified with plant size, providing a convenient, simple system for studying SDS (Méndez and Traveset 2003) without the confounding effects of flower and sexual organ number (potentially a trade-off with size), geitonogamy or the commitment of resources to leaves or floral nectar. Unlike nectar-producing species, in which the nectar reward may benefit both female and male function, this ambiguous sex allocation can be avoided in nectarless flowers of *P. hexandrum*. Otherwise, the use of the dry weight of sexual organs underestimates sex allocation at least in nectar-producing flowers.

To examine whether SDS occurs and varies in different populations of Himalayan mayapple, here we address three questions: (i) Does resource allocation to femaleness and maleness increase with plant size? (ii) Is sex allocation to female and male functions with plant size isometric or allometric? (iii) Does SDS vary across populations?

## Methods

### Study species and populations

*Podophyllum hexandrum* (Berberidaceae) is an alpine to subalpine perennial herb predominantly distributed in the Himalayan region at elevation from 2400 to 3400 m above sea level. Its perennating organ is the underground rhizome from which only one aerial stem develops after winter. The Himalayan mayapple blooms in May and the bowl-shaped flower generally lasts 4–5 days with six pink petals (Xiong *et al*. 2013). Its flower has no nectar or detectable odour. Two palmately divided leaves usually emerge after flower opening and rapidly extend in late May. Fruits are red and ripen in August. *Podophyllum hexandrum* is self-compatible and pollinated by solitary bees, and delayed self-pollination occurs at night through the closure of the petals (Xiong *et al*. 2013). We investigated one population at a field station in Shangri-La Alpine Botanical Garden and three populations near the garden (27°54′5″N, 99°38′17″E, 3300–3350 m a.s.l.), each with hundreds of individuals (Xiong *et al*. 2013).

### Sex allocation to floral organs

To evaluate the resource allocated to floral organs, we investigated two allocation currencies, biomass of sexual organs and gamete number. First, we randomly selected a total of 89 individuals with flower buds from four populations (Table 1, Miaopu, Titian, Sicun and Gongbincun). Flower buds were dissected into three parts: petals (attractive structure), stamens and pistil (sexual structure), which were desiccated at 50 °C overnight and then weighed to the nearest 0.01 g. To estimate pollen and ovule production per flower, we randomly collected 17, 18 and 17 flower buds (anthers undehisced) from the Miaopu, Sicun and Gongbincun populations. Anthers were split and pollen grains were suspended in 2 mL of water. Five drops of the pollen grain solution were counted under a microscope and the mean was calculated to estimate pollen production per flower (see Tang and Huang 2007). As an index of plant size, we measured the stem diameter at middle height of the stem for each individual (Niklas 1995) instead of collecting the plants, to avoid damaging the wild populations.

**Table 1.** Mean ± SD, range and sample size for stem diameters, dry weight of floral organs and gamete numbers in four populations of *P. hexandrum*, presenting with results of Pearson correlation analysis testing the relationship (*r*) between floral traits (floral organs mass and gamete numbers) and plant size.

	Variable	Mean ± SD	Range	*n*	*r*	*P*
Miaopu	Stem diameter (mm)	5.4 ± 1.0	3.6–7.6			
	Stamen (mg)	155.4 ± 44.1	68–270	22	0.729	0.000
	Pistil (mg)	84.2 ± 28.4	40–160	22	0.84	0.000
	Petal (mg)	199.8 ± 54.3	135–352	22	0.506	0.016
	Pollen number	77 866 ± 29 046	36 300–150 360			
	Ovule number	46 ± 21	22–119			
Titian	Stem diameter (mm)	5.0 ± 0.8	3.7–6.2			
	Stamen (mg)	138.5 ± 31.2	88–210	22	0.624	0.002
	Pistil (mg)	76.2 ± 21.7	47–125	22	0.692	0.000
	Petal (mg)	175.0 ± 39.7	90–245	22	0.657	0.000
	Pollen number					
	Ovule number					
Sicun	Stem diameter (mm)	4.8 ± 1.0	3.3–7.4			
	Stamen (mg)	151.8 ± 32.7	91–210	20	0.664	0.001
	Pistil (mg)	99.1 ± 31.1	41–166	20	0.747	0.000
	Petal (mg)	181.1 ± 47.5	105–255	20	0.598	0.005
	Pollen number	40 827 ± 20 330	10 020–78 420			
	Ovule number	55 ± 26	25–123			
Gongbingcun	Stem diameter (mm)	4.9 ± 0.8	3.7–6.9			
	Stamen (mg)	153.5 ± 24.9	103–209	25	0.390	0.054
	Pistil (mg)	96.4 ± 29.0	53–168	25	0.852	0.000
	Petal (mg)	208.9 ± 57.5	127–332	25	0.713	0.000
	Pollen number	34 150 ± 15 256	16 080–75 420			
	Ovule number	61 ± 24	31–107			

### Data analysis

Differences in average mass allocation to stamens and pistil per flower were tested by means of a paired samples *t*-test in four populations. Relationships between dry weight of floral organs and plant size were examined by Pearson correlation analysis. Similar statistical methods were used to analyse the relationships among different floral organ mass.

The allometric variation between pairs of floral organs (dry weight of stamens versus pistils, pollen versus ovule number, dry weight of attractive versus sexual structures) was examined following the procedures explained by Méndez (2001) who recommended a direct reduced major axis (RMA) regression of pairs of variables against each other for the high correlation among floral organs mass (*r* > 0.47; all *P* < 0.03, see Table 2). Prior to regression, pairs of variables were log_10_-transformed. If the resource allocation to the two sexes increases isometrically, the expected slope of the RMA regression of pairs of variables will be 1 (Sokal and Rohlf 1981). Any departure from expected values indicates preferential allocation. Departures from a slope value of 1 were tested by a one-sample *t*-test. To exclude the effect of population differences in the sex allocation pattern in *P. hexandrum*, the same analysis was conducted at species level with pooled data from the four populations together. The RMA regression analysis was performed using the RMA software (Bohonak and van der Linde 2004).

**Table 2.** Pearson correlation coefficients (significance) between dry weight of floral organs.

Population		Stamen	Pistil
Miaopu	Petal	0.472 (*P* = 0.026)	0.497 (*P* = 0.019)
	Stamen		0.730 (*P* = 0.000)
Titian	Petal	0.660 (*P* = 0.001)	0.818 (*P* = 0.000)
	Stamen		0.764 (*P* = 0.000)
Sicun	Petal	0.746 (*P* = 0.000)	0.769 (*P* = 0.000)
	Stamen		0.725 (*P* = 0.000)
Gongbincun	Petal	0.591 (*P* = 0.002)	0.886 (*P* = 0.000)
	Stamen		0.573 (*P* = 0.003)

## Results

Stem diameters varied over two times from 3.3 to 7.6, indicating obvious variation in plant size (Table 1). Flowers had a variable biomass (mg) of petals (from 90 to 352), stamens (68 to 270), pistils (40 to 168) and number of pollen (10 020 to 150 360) and ovules (22 to 123, Table 1). The dry weight of stamens per flower from four populations all significantly surpassed that of pistils (*t*_21_ = 11.012, *P* < 0.001; *t*_21_ = 14.422, *P* < 0.001; *t*_19_ = 9.942, *P* < 0.001 and *t*_24_ = 11.330, *P* < 0.001). The dry weight of floral organs (petal, stamen and pistil) correlated with each other (Table 2) and all were significantly positively related with plant size (but a marginally positive relationship of the stamen mass in Gongbincun population, Table 1).

The slope of the allometric relationship of biomass of stamens versus pistil did not significantly depart from 1 in Miaopu and Titian populations (Table 3), indicating an isometric allocation to maleness and femaleness with plant size, but the slopes in Sicun and Gongbingcun populations were significantly lower than 1 (Table 3), indicating a femaleness-bias allocation with plant size. When all populations were taken together, the pistils showed a significantly faster increase in allocation compared with the stamens (Table 3), suggesting a female-biased sex allocation with plant size in *P. hexandrum* at the species level. The slope of the relationship between pollen and ovule number did not significantly depart from 1 in all studied populations (Table 3), indicating an isometric allocation to sexual investment with plant size in *P. hexandrum* when pollen and ovule number are used as an indicator of male and female resource allocation. Taking all populations together, the slope did not significantly depart from 1 (Table 3), indicating an isometric growth of female and male gametes at the species level.

**Table 3.** Results of the RMA regression testing allometry (a slope significantly departed from 1) in different populations using different allocation currencies. Bold values indicated significant differences (*P* < 0.05).

Currency/population	*R*^2^	Slope ± SE	df	*t*	*P*
Stamen versus Pistil mass					
Miaopu	0.312	0.905 ± 0.168	21	−0.563	0.579
Titian	0.556	0.816 ± 0.122	21	−1.519	0.144
Sicun	0.663	0.677 ± 0.093	19	−3.488	**0.002**
Gongbincun	0.380	0.584 ± 0.096	24	−4.340	**<0.001**
Total	0.446	0.727 ± 0.058	88	−4.712	**<0.001**
Pollen versus ovule number					
Miaopu	0.278	0.974 ± 0.214	16	−0.122	0.904
Sicun	0.266	1.451 ± 0.311	17	1.450	0.165
Gongbincun	0.013	1.047 ± 0.269	16	0.175	0.863
Total	0.112	1.146 ± 0.153	51	0.954	0.344
Attractive versus sexual structures					
Miaopu	0.315	0.959 ± 0.178	21	−0.231	0.820
Titian	0.629	1.054 ± 0.144	21	0.375	0.711
Sicun	0.636	1.046 ± 0.149	19	0.309	0.761
Gongbincun	0.657	1.416 ± 0.173	24	2.405	**0.024**
Total	0.522	1.106 ± 0.082	88	1.293	0.200

The slope of the relationship between attractive and sexual structures did not significantly depart from 1 in three of the four studied populations except in Gongbincun population (Table 3), indicating an isometric allocation to attractive and sexual investment with plant size in most populations of *P. hexandrum*. Taking all populations together, the slope did not significantly depart from 1 (Table 3), indicating a proportionate resource allocation to attractive and sexual structures at the species level.

## Discussion

Overall, our investigation showed that relatively equal investment to both male and female function with plant size occurred in the animal-pollinated *P. hexandrum*; however, the pattern of SDS differed among populations and depended on the currency used to evaluate sex allocation (dry weight and gamete number). The dry weight of floral parts (stamens, pistils and petals) increased with plant size in all studied populations (Table 1). The rate of increase in allocation to stamens versus pistil showed no significant difference in two populations, while female-biased allocation with plant size was observed in the other two populations. When using pollen and ovule production as indicators of maleness and femaleness, isometric allocation to male and female function with plant size was observed in all examined populations of *P. hexandrum*. Furthermore, the dry weight of attractive versus sexual structures did not show significantly disproportionate increase with plant size.

Since sex in plants depends on individuals attaining a certain size threshold to produce flowers, a positive relationship between flower size and plant size is generally expected (Primack 1987) and was observed in numerous single-flowered species (*Erythronium japonicum*, Sakai 1998; two *Trillium* species, Wright and Barrett 1999; *Paeonia cambessedesii*, Méndez and Traveset 2003). The positive relationship between dry weight of floral parts and plant size in our study has provided the basis for further allometry analysis.

### Testing the hypothesis of SDS

It is usually expected that female function consumes more resources than male function. Thus, plants often tend to allocate more resources to male function in stressful environments with limited resources (Delph 1999). For example, in an aquatic, insect-pollinated monoecious herb *Sagittaria trifolia*, 48 % of ramets in the high-density population were male without female flowers (Han *et al*. 2011). Using the dry weight of stamens and pistils as the currency, *P. hexandrum* allocated more resource to stamens than pistils; however, a disproportionate female-biased increase with plant size was observed in Sicun and Gongbincun populations. This result is consistent with sex-differential resource cost hypothesis that female-biased allocation with plant size usually appears at a shaded site (Cao and Kudo 2008; Torices and Méndez 2011).

The theory of sex allocation proposes that the relative quantities of resource allocated to male and female function depend on the fitness returns from the two sexes (Charnov 1982; de Jong and Klinkhamer 2005). However, in many animal-pollinated plants, the male fitness return decelerated more than the female fitness return, resulting in a female-biased allocation for local mate competition (Lloyd and Bawa 1984) and geitonogamy level (Harder and Barrett 1995; de Jong 2000). In two *Trillium* species, both biomass and gamete production were observed to be female-biased allocation with increasing plant size, which was partly attributed to reducing local mate competition because of low pollinator visitation rates, frequent pollinator limitation and the following high levels of correlated paternity in these two species (Wright and Barrett 1999). The two of four populations of *P. hexandrum* showed isometric allocation to male and female investment using sexual organ mass and three populations showed isometric sex allocation using gamete number to evaluate sexual investment (Table 3). Capability of automatic self-pollination contributed to seed production and no significant self-incompatibility was observed in *P. hexandrum* (Xiong *et al*. 2013). Therefore, the male fitness return in *P. hexandrum* may decelerate much more slowly than expected for two reasons: (i) the low pollen loss during pollen transfer (pollen could be deposited directly onto stigmas automatically) and (ii) the deleterious effect of pollen discounting is minor because of no significant self-incompatibility (Xiong *et al*. 2013). These may explain the isometric sex allocation in *P. hexandrum*, in contrast to the common pattern of SDS that female-biased allocation increasing with plant size as observed in the two *Trillium* species (Wright and Barrett 1999). Similarly, pollen and ovule production per flower increased isometrically with plant size was observed in predominantly selfing *Clarkia* species, suggesting that obligate selfers gain fitness equally through male and female function (Delesalle and Mazer 2009). It is interesting to know whether American mayapple *Podophyllum peltatum* which is basically self-incompatible (Crants 2008) evolves female-biased allocation with plant size.

### Attractive versus sexual structures

Attractive structures have been considered to affect the resource allocation within flowers. Previous studies suggested that cosexuality should be stabilized by high allocation to attractive structures under pollen-limited environment (Charlesworth and Charlesworth 1987; Charlesworth and Morgan 1991). Méndez (2001) found a disproportionately increased allocation to attractive structures (spathe) in *Arum italicum*. In this species, an increasing spathe length significantly increased the number of insects trapped in the inflorescences, potentially beneficial for pollination success. In two related heterantherous herb, *Monochoria korsakowii* is mainly outcrossing and *Monochoria vaginalis* is a predominant selfer. Tang and Huang (2007) observed that a disproportional reduction in pollen production of the feeding anthers in the selfing species, suggesting that enhanced allocation to attractive structures in outcrossing species may facilitate pollen dispersal. Torices and Méndez (2011) also observed a preferred resource allocation to attractive structures at a shaded site of a pollen-limited species *T. farfara*, while at an open site allocation to attractive and sexual structures was found to be isometric. In our study, most populations showed isometric allocation to attractive and sexual structures (Table 3), suggesting no selection for an increased investment to attractive structures in *P. hexandrum* which has evolved automatically selfing.

### Organ mass versus gamete number

If the resource allocated to femaleness and maleness increases linearly with the number of gametes produced, the same SDS pattern should be observed using either dry weight of sexual organs or the number of gametes to evaluate sex allocation. However, we observed contrasting sex allocation patterns in Sicun and Gongbincun populations using different allocation currencies (dry weight and gamete number). The contrasting sex allocation patterns when different allocation currencies are used might imply a trade-off between pollen size and number in Sicun and Gongbingcun population. Such a trade-off within species has been found in numerous genera (see Vonhof and Harder 1995). The trade-off between gamete size and number may potentially constrain the use of pollen and ovule number as allocation currency compared with the use of biomass. In addition, various researchers have emphasized resource costs of female reproduction at fruiting (Lloyd and Bawa 1984; Bickel and Freeman 1993; Day and Aarssen 1997), which could not be simply evaluated by ovule production at flowering, especially there are trade-offs between the number and size of ovules and seeds. However, gamete production is essential to examine hypotheses of local mate/resource competition (Lloyd and Bawa 1984) which predict that increased pollen number would decrease the opportunity of a given pollen fertilizing an ovule, and fewer ovules would decrease the competition between sib offsprings under resource limitation condition. Varied sex allocation pattern was observed in a monoecious herb *S. trifolia*, in which sex allocation was isometric when flower number of the two sexes was used but allometric when biomass was used as allocation currency (Han *et al*. 2011).

## Conclusions

Our investigation indicated that sex allocation to female and male functions was generally isometric in Himalayan mayapple but the SDS pattern varied across populations. It is thus necessary to consider the effect of number and size when using gamete or structure number as a currency of sex allocation to the greatest degree possible. Further studies on sexual allocation will benefit from measurements involving different allocation currencies (Goldman and Willson 1986; Ashman and Baker 1992; Méndez and Traveset 2003).

## Sources of Funding

This work was supported by the National Science Foundation of China (no. 31270281).

## Contributions by the Authors

Y.-Z.X. and M.X. collected data from the field populations. Y.-Z.X. and S.-Q.H. wrote the manuscript. All authors contributed in experimental design, data analysis and commented the manuscript.

## Conflict of Interest Statement

None declared.

## References

[PLV139C1] Ashman TL, Baker I 1992 Variation in floral sex allocation with time of season and currency. Ecology 73:1237–1243. 10.2307/1940672

[PLV139C2] Bickel AM, Freeman DC 1993 Effects of pollen vector and plant geometry on floral sex ratio in monoecious plants. American Midland Naturalist 130:239–247. 10.2307/2426124

[PLV139C3] Bohonak AJ, van der Linde K 2004 RMA: software for reduced major axis regression, Java version. http://www.kimvdlinde.com/professional/rma.html (15 October 2015).

[PLV139C4] Cao GX, Kudo G 2008 Size-dependent sex allocation in a monocarpic perennial herb, *Cardiocrinum cordatum* (Liliaceae). Plant Ecology 194:99–107.

[PLV139C5] Charlesworth D, Charlesworth B 1987 The effect of investment in attractive structures on allocation to male and female functions in plants. Evolution 41:948–968. 10.2307/2409184 28563419

[PLV139C6] Charlesworth D, Morgan MT 1991 Allocation of resources to sex functions in flowering plants. Philosophical Transactions of the Royal Society of London B: Biological Sciences 332:91–102. 10.1098/rstb.1991.0036

[PLV139C7] Charnov EL 1982 The theory of sex allocation. Princeton, NJ: Princeton University Press.

[PLV139C8] Crants JE 2008 Pollination and pollen limitation in mayapple (*Podophyllum peltatum* L.), a nectarless spring ephemeral. PhD Thesis, University of Michigan.

[PLV139C9] Day T, Aarssen L 1997 A time commitment hypothesis for size-dependent gender allocation. Evolution 51:988–993. 10.2307/2411173 28568576

[PLV139C10] de Jong TJ 2000 From pollen dynamics to adaptive dynamics. Plant Species Biology 15:31–41. 10.1046/j.1442-1984.2000.00028.x

[PLV139C11] de Jong TJ, Klinkhamer PGL 2005 Evolutionary ecology of plant reproductive strategies. Cambridge: Cambridge University Press.

[PLV139C12] Delesalle VA, Mazer SJ 2009 Size-dependent pollen: ovule ratios and the allometry of floral sex allocation in *Clarkia* (Onagraceae) taxa with contrasting mating systems. American Journal of Botany 96:968–978. 10.3732/ajb.0800039 21628249

[PLV139C13] Delph LF 1999 Sexual dimorphism in life history. In: Geber MA, Dawson TE, Delph LF, eds. Gender and sexual dimorphism in flowering plants. New York: Springer, 149–173.

[PLV139C14] Goldman DA, Willson MF 1986 Sex allocation in functionally hermaphroditic plants: a review and critique. The Botanical Review 52:157–194. 10.1007/BF02861000

[PLV139C15] Han B, Wang XF, Huang SQ 2011 Production of male flowers does not decrease with plant size in insect-pollinated *Sagittaria trifolia*, contrary to predictions of size-dependent sex allocation. Journal of Systematics and Evolution 49:379–385. 10.1111/j.1759-6831.2011.00141.x

[PLV139C16] Harder LD, Barrett SCH 1995 Mating cost of large floral displays in hermaphrodite plants. Nature 373:512–515. 10.1038/373512a0

[PLV139C17] Klinkhamer PGL, de Jong TJ, Metz H 1997 Sex and size in cosexual plants. Trends in Ecology and Evolution 12:260–265. 10.1016/S0169-5347(97)01078-1 21238063

[PLV139C18] Lloyd DG, Bawa KS 1984 Modification of the gender of seed plants in varying conditions. Evolutionary Biology 17:255–338. 10.1007/978-1-4615-6974-9_6

[PLV139C19] Méndez M 2001 Sexual mass allocation in species with inflorescences as pollination units: a comparison between *Arum italicum* and *Arisaema* (Araceae). American Journal of Botany 88:1781–1785. 10.2307/3558353 21669610

[PLV139C20] Méndez M, Traveset A 2003 Sexual allocation in single-flowered hermaphroditic individuals in relation to plant and flower size. Oecologia 137:69–75. 10.1007/s00442-003-1319-z 12844252

[PLV139C21] Niklas KJ 1995 Size-dependent allometry of tree height, diameter and trunk-taper. Annals of Botany 75:217–227. 10.1006/anbo.1995.1015

[PLV139C22] Primack RB 1987 Relationships among flowers, fruits, and seeds. Annual Review of Ecology and Systematics 18:409–430. 10.1146/annurev.es.18.110187.002205

[PLV139C23] Sakai S 1998 Female-biased sex allocation in relation to growth rate of fruit in *Erythronium japonicum*. Oecologia 117:391–395. 10.1007/s004420050672 28307918

[PLV139C24] Sokal RR, Rohlf FJ 1981 Biometry, 2nd edn San Francisco, CA: W. H. Freeman.

[PLV139C25] Tang LL, Huang SQ 2007 Evidence for reductions in floral attractants with increased selfing rates in two heterandrous species. New Phytologist 175:588–595. 10.1111/j.1469-8137.2007.02115.x 17635233

[PLV139C26] Torices R, Méndez M 2011 Influence of inflorescence size on sexual expression and female reproductive success in a monoecious species. Plant Biology 13:78–85. 10.1111/j.1438-8677.2009.00292.x 21134090

[PLV139C27] Vonhof MJ, Harder LD 1995 Size-number trade-offs and pollen production by papilionaceous legumes. American Journal of Botany 82:230–238. 10.2307/2445530

[PLV139C28] Wright SI, Barrett SCH 1999 Size-dependent gender modification in a hermaphroditic perennial herb. Proceedings of the Royal Society B: Biological Sciences 266:225–232. 10.1098/rspb.1999.0626

[PLV139C29] Xiong Y-Z, Fang Q, Huang S-Q 2013 Pollinator scarcity drives the shift to delayed selfing in Himalayan mayapple *Podophyllum hexandrum* (Berberidaceae). AoB PLANTS 5: plt037; 10.1093/aobpla/plt037 10.1093/aobpla/plt037

